# Rapid prototyping of models for COVID-19 outbreak detection in workplaces

**DOI:** 10.1186/s12879-023-08713-y

**Published:** 2023-10-23

**Authors:** Isobel Abell, Cameron Zachreson, Eamon Conway, Nicholas Geard, Jodie McVernon, Thomas Waring, Christopher Baker

**Affiliations:** 1 School of Mathematics and Statistics, The University of Melbourne, Melbourne, Australia; 2 Melbourne Centre for Data Science, The University of Melbourne, Melbourne, Australia; 3 School of Computing and Information Systems, The University of Melbourne, Melbourne, Australia; 4 Walter and Eliza Hall Institute of Medical Research, Melbourne, Australia; 5 Peter Doherty Institute for Infection and Immunity, The University of Melbourne and the Royal Melbourne Hospital, Melbourne, Australia; 6 Centre for Epidemiology and Biostatistics, Melbourne School of Population and Global Health, The University of Melbourne, Melbourne, Australia; 7 Murdoch Children’s Research Institute, The Royal Children’s Hospital, Melbourne, Australia; 8 Centre of Excellence for Biosecurity Risk Analysis, The University of Melbourne, Melbourne, Australia

**Keywords:** Infectious disease modelling, Decision making

## Abstract

Early case detection is critical to preventing onward transmission of COVID-19 by enabling prompt isolation of index infections, and identification and quarantining of contacts. Timeliness and completeness of ascertainment depend on the surveillance strategy employed. This paper presents modelling used to inform workplace testing strategies for the Australian government in early 2021. We use rapid prototype modelling to quickly investigate the effectiveness of testing strategies to aid decision making. Models are developed with a focus on providing relevant results to policy makers, and these models are continually updated and improved as new questions are posed. Developed to support the implementation of testing strategies in high risk workplace settings in Australia, our modelling explores the effects of test frequency and sensitivity on outbreak detection. We start with an exponential growth model, which demonstrates how outbreak detection changes depending on growth rate, test frequency and sensitivity. From the exponential model, we learn that low sensitivity tests can produce high probabilities of detection when testing occurs frequently. We then develop a more complex Agent Based Model, which was used to test the robustness of the results from the exponential model, and extend it to include intermittent workplace scheduling. These models help our fundamental understanding of disease detectability through routine surveillance in workplaces and evaluate the impact of testing strategies and workplace characteristics on the effectiveness of surveillance. This analysis highlights the risks of particular work patterns while also identifying key testing strategies to best improve outbreak detection in high risk workplaces.

**Supplementary Information:**

The online version contains supplementary material available at 10.1186/s12879-023-08713-y.

## Introduction

Rapid prototype modelling is a model development approach that aims to provide rapid insights while laying the foundations for more detailed modelling [1]. Models developed should be simple yet still convey the complexities and subtleties of a problem to decision makers [2]. With each iteration, models are updated to reflect new scenarios arising from revised information and questions [1, 3]. Rapid response modelling has been crucial in developing policy for COVID-19, allowing key hypotheses and assumptions to be tested in real-time as public health policy is implemented [4]. In this work we demonstrate how we used rapid prototyping to develop models of workplace testing strategies to guide the Australian Government response to COVID-19 in 2021.

For countries with COVID-19 management policies aimed at zero community prevalence, accurate and timely case detection is necessary to suppress outbreak. In 2020, Australia was one such country, where the official COVID-19 policy was “strong suppression” until vaccines were widely distributed throughout 2021. Before widespread vaccination, COVID-19 spread was prevented by proactive management of borders, active case finding and follow up with strict isolation and quarantine requirements for cases and contacts, and through liberal access to PCR testing in both high risk settings and the general community. Arriving international travellers posed the greatest risk of imported infection, leading to mandatory hotel quarantine arrangements on the 28^th^ of March that were maintained through to late 2021 [5]. Testing was part of a number of infection prevention and control interventions aimed at minimising the risk of community outbreaks. However, repeated SARS-CoV-2 incursions to the community, despite stringent arrivals procedures, prompted the redesign of testing strategies for travellers and workers in quarantine settings.

Outbreak detection via testing is influenced by multiple factors including test performance (sensitivity and specificity) and test frequency. PCR tests are seen as the ‘gold standard’ in testing practices, although their performance varies over the course of the infection [6], with many individuals remaining PCR-positive after they are no longer infectious. Furthermore, the slow turnaround time for PCR tests (typically days) can delay outbreak detection [6]. While Rapid Antigen Tests (RATs) typically have a turnaround time of 15 minutes, they have lower sensitivity and specificity than PCR tests [7]. Similar to PCR tests, test sensitivity varies depending on the stage of infection, and whether infection is symptomatic or asymptomatic [7, 8]. Existing work suggests that these issues can be overcome by increasing the frequency of testing, implying that RATs can still be practically useful despite their limitations [9, 10].

In early 2021, Australia was seeking to improve testing strategies in workplaces, with the primary objective of detecting new outbreaks quickly. However, the differences in performance characteristics between different tests made it challenging to develop a robust workplace testing strategy. In addition to test performance, there were also questions about how the emergence of variants of concern would affect outbreak detection, prompted by the emergence of the Alpha variant, which was both more transmissible and more pathogenic than antecedent viruses [11].

In this work, we describe how we used a rapid prototyping approach to provide timely advice on the principles underlying a robust workplace testing policy. That is, we estimated the sensitivity of alternative surveillance strategies using models of differing granularity. The first is the ‘exponential model’, which we developed to give timely insight into testing efficacy. The model is defined by exponential growth of disease prevalence in the workplace, and allows us to quickly understand the interactions between test sensitivity, frequency and growth rate ($R_{\text {eff}}$) on outbreak detection. The second model developed is an agent based model (ABM) which allows us to investigate the interaction between scheduled testing frequency and shift work patterns in determining the overall sensitivity of the surveillance system. The ABM builds on assumptions made by the rapidly developed exponential model to probe and update its results to answer new, emerging questions for an updated situation (i.e. intermittent work scheduling). These two models were developed as tools to provide answers to specific questions posed by policy makers about designing effective testing practices in workplaces. The ABM presented here was subsequently updated to suit new questions from decision-makers concerning the impact of quarantine on outbreak management [12].

## Modelling

We present our two models and results in the sequence they were developed, starting with the exponential model, which we use to explore the effects of growth rate ($R_{\text {eff}}$), test sensitivity and test frequency on the probability of detecting outbreaks in workplaces. We then consider the Agent Based Model (ABM), using it to probe the results of the exponential model and also to explore the impact of more complex work schedules on the probability of outbreak detection.

When these models were developed, Australia had very few cases of COVID-19 and decision-makers were concerned with outbreaks stemming from single positive cases. As such, our modelling is framed around how quickly *any* case could be detected in “high-risk” workplaces. In particular, we calculate the probability an outbreak is detected within a week. This timeframe was chosen in collaboration with decision-makers.

For both the exponential model and the ABM, we define an outbreak occurring when at least one individual is infected. An outbreak is detected when a positive case is identified, either through testing or symptom onset.

### Exponential model

Our initial model for the probability of detecting a COVID-19 outbreak in a workplace assumes exponential growth of active cases. In the exponential model, disease prevalence on day $i+1$ (denoted $P_{i+1}$) is given by:

1 $$\begin{aligned} P_{i+1} = P_i(R_{\text {eff}})^{\frac{1}{g}}, \end{aligned}$$

where $P_i$ is the disease prevalence on day *i*, *g* is the generation interval and $R_{\text {eff}}$ is the effective reproduction number. We assume a generation interval of 4.7 days, and that an outbreak begins on the first day with one active infection ($P_0 = 1$)[13]. With these assumptions, we calculate the expected number of active cases through time under different values of $R_{\text {eff}}$. We assume all infections are due to contacts within the workplace, that is there are no cases imported into the workplace other than the initially seeded infection. Note that this model does not distinguish between symptomatic and asymptomatic infection. Further details about the exponential model are provided in the Supplementary material.

Using our model of prevalence, we then calculate the probability of detecting at least one case within a week under different testing strategies. We vary testing strategies by considering different test sensitivities and test frequency, i.e. the number of days per week testing occurs. In our results we consider scenarios where testing occurs once per week, three times per week and daily. We assume that on days when testing occurs, the entire workforce is tested. This testing framework was chosen to suit workplaces with high importation risk, such as quarantine hotels.

The one-week detection probability is defined as the probability an infected individual returns a positive test within a week of the initial infection. Let $P_i$ be the prevalence on testing day *i*, and *s* the test sensitivity. In our model, we make the simplifying assumption that each test is independent and test sensitivity a) does not vary between people and b) does not varying across a person’s infectious period. Furthermore, we assume tests have $100\%$ specificity. The probability an outbreak is *not* detected on day *i* (given everyone is tested) is:

2 $$\begin{aligned} Pr(\text {no detection on day { i}})&= Pr(\text {all infected people test negative})\end{aligned}$$

3 $$\begin{aligned}&= (1-s)^{P_i}. \end{aligned}$$

Let *T* be the set of testing days in a given week, i.e. days where everyone is tested. The probability of detection in a week is therefore given by:

4 $$\begin{aligned} Pr(\text {detection in a week})&= 1- Pr(\text {no detection on all testing days})\end{aligned}$$

5 $$\begin{aligned}&= 1 - \prod _{i \in T} Pr(\text {no detection on day} i)\end{aligned}$$

6 $$\begin{aligned}&= 1 - \prod _{i \in T} (1-s)^{P_i}. \end{aligned}$$

### Exponential model results

**Fig. 1:**
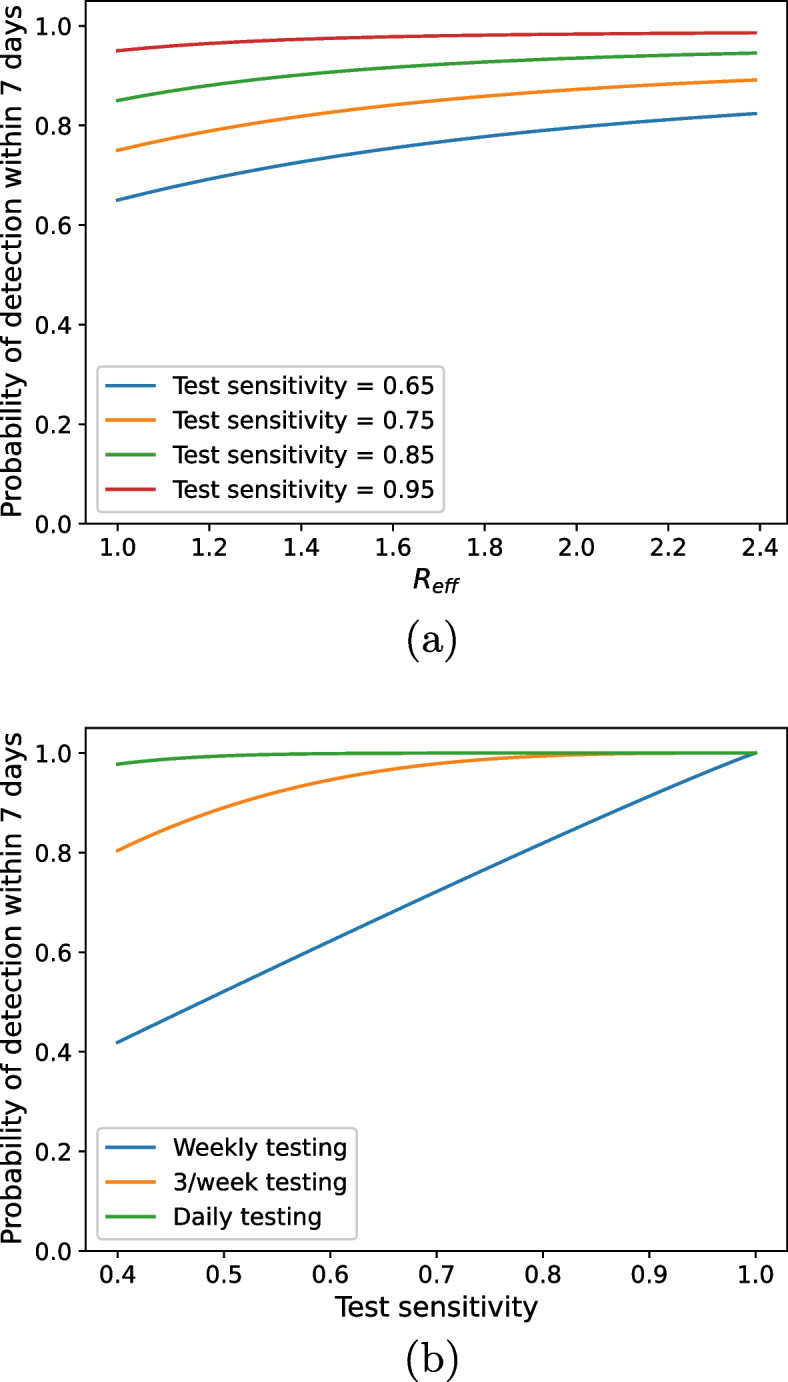
Probability of detection within a week using the exponential model as we vary growth rate ($R_{\text {eff}}$) and test sensitivity (**a**), and test sensitivity and testing schedule (**b**). We assume a generation interval of 4.7 days and a workplace size of 50 people for both **a** and **b**. For **a** we assume testing occurs only once per week and for **b** we assume a conservative growth rate of $R_{\text {eff}} = 1.1$

The probability of detection within a week increases with both test sensitivity and $R_{\text {eff}}$ (Fig. 1a). With testing occurring once per week, there is a large difference between whether there is low (0.65) or high (0.95) test sensitivity. However, the difference in the probability of outbreak detection between low and high sensitivity decreases with increasing $R_{\text {eff}}$. As $R_{\text {eff}}$ increases the outbreak spreads faster, meaning more infected people are tested within a week, increasing the likelihood that at least one is detected.

Increasing test sensitivity and frequency both increase the probability of outbreak detection within a week (Fig. 1b). Most notably, daily testing results in a high probability of detection within a week ($>95\%$), for all test sensitivities. This demonstrates that low-sensitivity tests, such as RATs, are still useful for outbreak detection — their shortcomings can be overcome by more frequent testing.

### Agent based model

We develop an agent based model (ABM) to represent additional complexities of the workplace setting not captured in the exponential model. The ABM incorporates further complexity and allows us to ask more detailed questions about workplace testing. We start by setting up the ABM using the same set of assumptions as the exponential model. In replicating the exponential model results using the ABM, we can be confident that the ABM generalises the earlier results.

Each agent in our model follows an *susceptible*–*exposed*–*infectious*–*recovered* disease progression. Agents begin each simulation *susceptible*, and once infected become *exposed*. An exposed agent is neither infectious nor detectable. Exposed agents will transition to an *incubating* phase, where they become both infectious and detectable by testing. In the incubating phase, agents are either *symptomatic* or *asymptomatic*. There are no differences in transmission or infection period between symptomatic or asymptomatic individuals, but it allows us to track symptom onset. Both symptomatic and asymptomatic infections are detectable by testing, but symptomatic infection is also detected at the moment of symptom onset. When their infection ends, agents become *recovered*, and immune to reinfection. Further modelling details, including parameter values, can be found in the Supplementary material.

For each simulation, outbreaks are seeded by a single infection in the workplace. That is, one randomly selected agent starts “exposed” for each simulation. As for the exponential model, there are no infections imported into the workplace other than the initially seeded infection. An outbreak is detected at symptom onset of an infected person or when a positive test result is returned. As for the exponential model, we assume test sensitivity is constant across all people and across infectious periods, and that the tests have 100% specificity. We assume that two thirds of infectious people are symptomatic. For each model simulation, we estimate the probability of detecting at least one case within a week over 5000 simulation instances, each of which is subject to stochastic variation. We calculate this probability as the proportion of simulation instances resulting in outbreak detection within a week.

When considering the ABM under the exponential model assumptions, we assume there is no latency period and no detection via symptom onset. As there is no difference in transmission or infection period between symptomatic and asymptomatic infections, we can simulate the ABM with no detection via symptom onset by considering all infections to be asymptomatic. This is simply for ease of calculation and does not imply in this case we only consider asymptomatic testing.

We define test frequency by specifying the number of testing days per week. As in the exponential model, on days when testing occurs, everyone attending the workplace is tested.

To extend the exponential model results, we consider the effects of intermittent work schedules on outbreak detection. We define these work schedules by specifying the proportion of the workplace working 1, 3, 5 or 7 days a week. Employees are then randomly assigned work days in accordance with the number of days they are scheduled to work. We assume people are only tested at work, but outbreaks may be detected through symptom onset at any time, regardless of whether the unwell individual is present in the workplace.

### Agent based model results

In this section, we present results from the ABM. We start by reproducing results from the exponential model to see how the ABM aligns with previous results. We then update our assumptions to consider the impact of intermittent workplace attendance on the probability of outbreak detection within a week of the introduction of the virus.

#### Comparing the exponential and agent based models

**Fig. 2:**
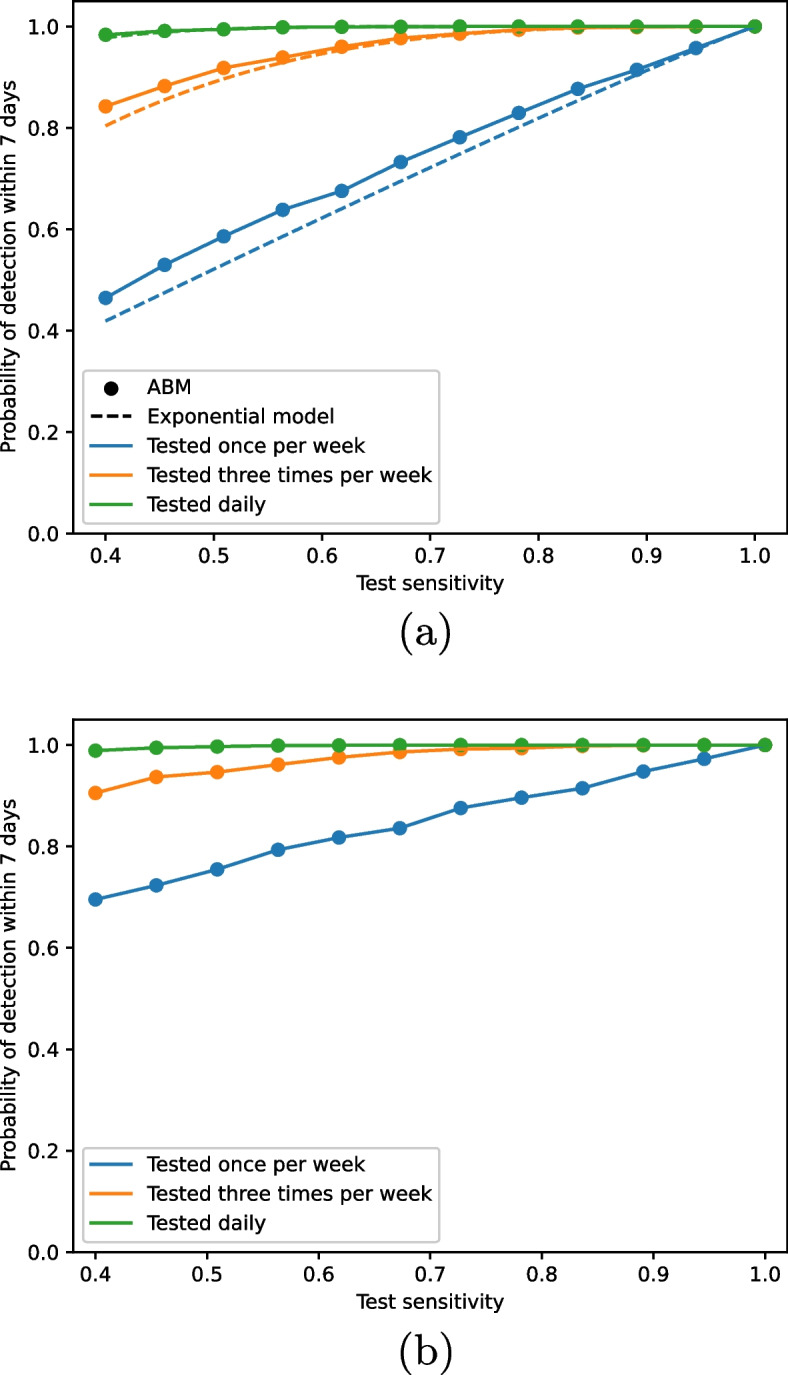
Probability of detection within a week as calculated by the exponential and agent based models as we vary growth rate, test sensitivity and test frequency. **a** compares the ABM results to the exponential model under the same assumptions, i.e. no latent infection period, no asymptomatic infection and no detection via symptom onset. **b** shows the ABM results under different assumptions to the exponential model, i.e. a latent infection period (1 day), asymptomatic infection and detection via symptom onset

In line with the process of rapid prototyping, Fig. 2 compares the exponential model behaviour to that of the ABM. Under identical sets of assumptions, the ABM results closely follow those of the exponential model (Fig. 2a), although the ABM produces slightly more optimistic estimates of detection probability. However, when we change assumptions of the ABM (Fig. 2b), the results begin to diverge. Under the new assumptions, the results from the ABM produce much higher probabilities of outbreak detection than the exponential model. This is explained by the additional mode of detection, by symptom onset. The additional assumptions we use here cannot be built into the exponential model due to its simplicity, so the development of the ABM allows us to explore the impact of these infection and testing characteristics on outbreak detection.

#### Intermittent workplace attendance

A strength of the ABM is that it can be used to explore the implications of more complex patterns of workplace attendance. We introduce an intermittent work schedule, where some proportion of workers work 1, 3, 5 and 7 days a week. Analogously to the testing assumptions of the exponential model, we assume that when testing occurs, everyone in the workplace on that day is tested. We consider the following intermittent work schedules defined by the proportion of the workforce working 1, 3, 5 or 7 days a week:

1. $$100\%$$ 7 days/week,
2. $$100\%$$ 5 days/week,
3. $60\%$ 5 days/week, $40\%$ 3 days/week,
4. $60\%$ 5 days/week, $30\%$ 3 days/week, $10\%$ 1 day/week.

**Fig. 3:**
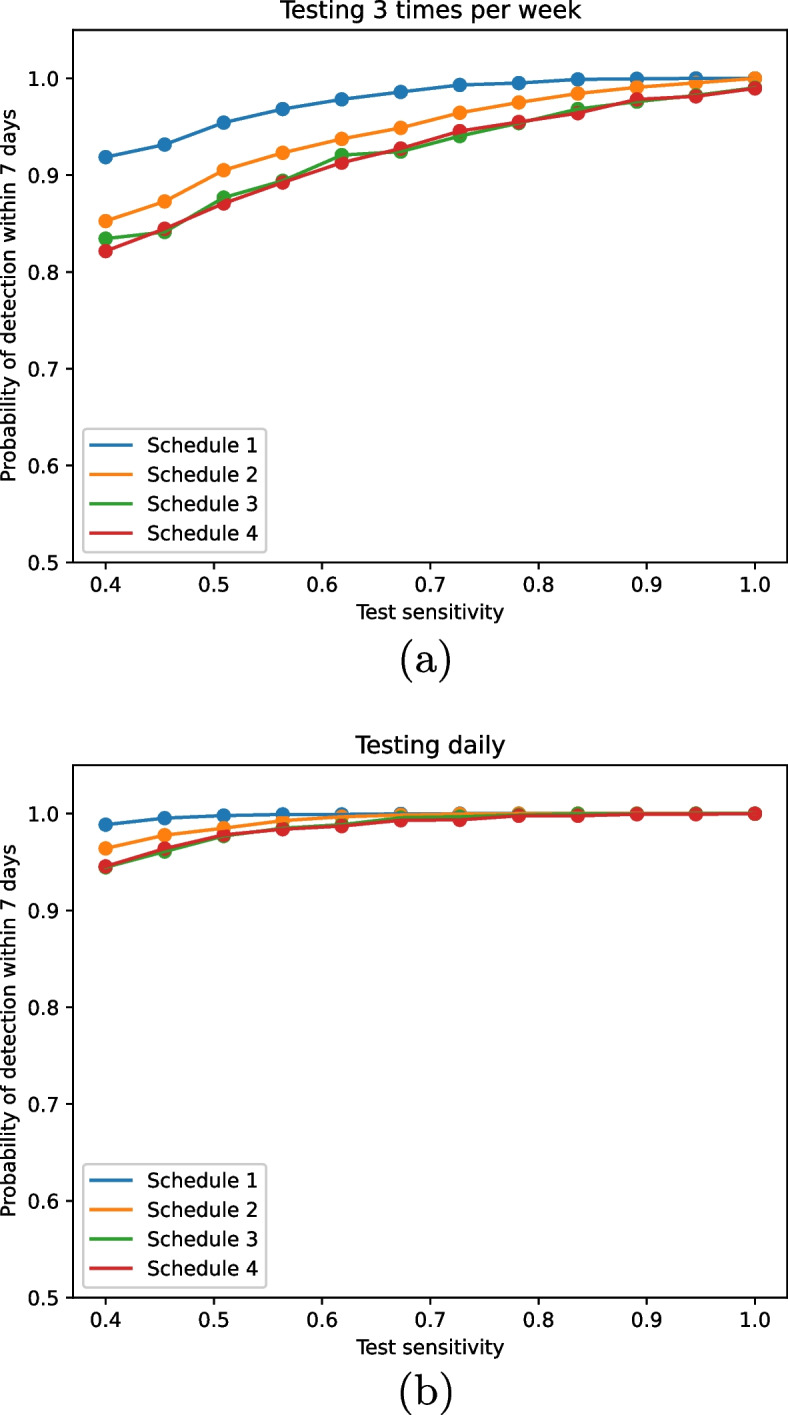
Probability of detecting an outbreak within a week under various intermittent working schedules as we vary test sensitivity and frequency for **a** testing three times per week and **b** testing daily. See text for details of testing schedules

As observed in the exponential model, increasing test frequency and sensitivity increases the probability of detecting an outbreak (Fig. 3). In a similar way, the detection probability is higher when employees work more frequently. Notably, even for a sparse work schedule, low test sensitivity can be compensated for with higher testing frequency. If we increase test frequency, employees are more likely to be tested in a given week as they are more likely to be at work on a testing day. This compounds the benefits of frequent testing observed in the exponential model.

The ABM includes the assumption that outbreaks can be detected by symptom onset, which imposes an upper bound on the time to detection. As symptom onset can occur outside the workplace, outbreaks can be detected even when an infected worker is at home. This bound increases the seven-day detection probability compared to the exponential model.

## Discussion

Pandemic policies need to be adaptable in the face of emerging epidemic intelligence, including changes in circulating pathogen characteristics, host-pathogen interactions and diagnostic modalities [14]. By reassessing and updating our prototype models to examine new questions posed, we can aid decision makers by supporting evidence–based decisions as new scenarios arise. The development of the exponential model and the ABM demonstrates how a rapid prototyping approach is useful for informing disease-management policy. While simple by design, the exponential model was quickly able to show that lower sensitivity tests can be useful when combined with high frequency testing, and that variants with higher $R_0$ may be more readily detected in outbreak settings than less transmissible strains.

Answering these early questions naturally led to more nuanced questions, and we developed our second model in response to this. The ABM explores how the interaction between shift patterns and routine surveillance testing frequency determines the effective testing rate across the workforce. In our model, a high symptomatic proportion ($>60\%$) and perfect compliance with testing requirements mitigated identified risks associated with gaps in surveillance due to non-work days. These assumptions were valid in context of an unvaccinated population, circulation of the Alpha variant and strict public health orders mandating testing requirements. Assumptions must be updated in light of population and pathogen characteristics. For example in the case of COVID-19, by late 2022 a much lower symptomatic proportion would be expected given high levels of population vaccine coverage in Australia and emergence of the less pathogenic Omicron variant.

While Australia’s COVID-19 risk environment is continually evolving, both our modelling and the rapid prototyping framework can still provide useful insights. In contrast to the COVID-19 landscape in early 2021, Australia now has widespread COVID-19 transmission, meaning there are fewer workplaces where we actively seek to detect new outbreaks [15]. However, there remain workplaces, for example aged care facilities, where there is a high chance of severe outcomes from COVID-19, and so we would still seek to detect new outbreaks quickly to put in place mitigation measures. Furthermore, our models are fairly general, so our results are not specific to COVID-19. The exponential model assumes that we are aiming to detect something that is increasing in prevalence through time, and the only COVID-19 specific assumption is about the generation interval. Similarly, the ABM is quite general and these models could be readily adapted for other pathogens.

To answer questions around testing strategies in workplaces, our modelling aimed to model the decision problem at hand rather than simulate an outbreak as realistically as possible. When designing models using a rapid prototyping approach, we cannot guarantee that the model will be suitable *outside* the scope of the question it is designed to answer. It is not necessary that the models we use are the “best” models of the system, but instead that they are suitable for the given questions. For example, our models parsimoniously consider populations to be homogeneous when we know characteristics such as age play a crucial role in infection dynamics for COVID-19 [16]. In particular, these heterogeneities can impact test sensitivity both across a population and across an individual’s infectious period. While it is important to consider these complexities, they were not crucial to answering questions from decision-makers. Developing models at each stage of a rapid prototyping approach requires weighing model limitations against usefulness for answering questions. However, results from models with different structures will always differ due to the implicit assumptions embedded in each model, e.g. implied generation time distributions [17]. Comparing models under the same assumptions allows us to assess structural uncertainty. That is, we can ensure new results are the result of updated assumptions, rather than changed model structure or implementation.

Effective modelling to support decision-making relies on communication with, and feedback from, decision-makers. While we have discussed iteration of model design, continuously seeking feedback from decision-makers on aspects of communication and study design is also crucial to successful rapid prototyping. For example, feedback from decision makers on model visualisations and results presented can help improve how modelling is communicated in the next iteration. Furthermore, consistent communication with decision-makers ensures modelling aspects, such as target measures and model assumptions, best align with the context of the problem considered. Incorporating feedback on both modelling and its communication from decision-makers helps update modelling in the next iteration to better suit questions posed.

Code sharing and modelling workflows remain challenging in rapidly evolving situations. During these projects, code was developed on internal, shared version–controlled repositories. The code has been re-written for clarity and made available in a public repository.

## Conclusion

Our study shows the utility of taking a rapid prototyping approach to model development in epidemiology, starting by developing simple models and then building in additional complexity. Rapid prototyping has been used effectively for environmental management as part of Structured Decision Making approaches [1, 3], but has not been used formally in epidemiology. Like ecological fields, epidemiology is well-suited for rapid prototyping due to its range of well-known simple models, e.g. SIR type models. In our example of workplace outbreak detection, the exponential model results provide a pessimistic estimate for the probability of outbreak detection. With updated information, the ABM provides a more realistic estimate of the probability of outbreak detection. Here, rapid prototyping allows us to provide a quick, conservative estimate to policy makers which can then be updated as more information becomes available.

The modelling described here forms one component of a larger body of work supporting COVID-19 decision making in Australia. Our modelling was commissioned through the Australian Government Office of Health Protection by the Public Health Laboratories Network (PHLN). PHLN is an advisory group providing technical advice to the Australian Government about public health microbiology and communicable disease control [18]. Prior to this work, modelling was used to investigate the test turnaround time and targeting of testing of PCR tests to reduce COVID-19 transmission [19]. This then prompted questions about testing strategies incorporating RATs in high-risk workplaces that informed the work presented in this paper. Our modelling was presented to PHLN and the Australian Health Protection Principal Committee (AHPPC) in February 2021 to inform overarching national minimum guidelines for workplace testing [20]. Following the rapid prototyping framework, our ABM led to subsequent modelling of the impact of quarantine on outbreak management [12]. This model builds on the work presented here, incorporating more complex considerations such as variable test sensitivity over the course of an individual’s infectious period and the impact of timely quarantine on outbreak probability. Alongside the quarantine model, further related modelling focused on investigating testing strategies for specific settings such as schools [21] and Indigenous communities [22].

The COVID-19 pandemic has highlighted the importance of model-generated evidence in decision making. With a short time-frame in which to answer questions, and a rapidly changing set of circumstances, flexible models which can be updated to new questions have an important role. The rapid prototyping process we describe is well suited to informing policy in a quickly evolving situation. However, there remain practical challenges such as ensuring team members involved can contribute to reproducible and version controlled coding within strict timeframes. While the importance of gaining quick insights for policy is clear, an additional benefit is that rapid prototyping models provide direction for development of more complex models. Simple models can provide useful insights to inform strategic thinking, and more detailed models are able to incorporate important real world complexities to refine tactics for surveillance and response.

### Supplementary Information

**Additional file 1.**

## Data Availability

The code used to generate all results in this manuscript is available online https://github.com/iabell/rapid_prototyping_COVID_19.

## References

[1] Garrard GE, Rumpff L, Runge MC, Converse SJ, Bunnefeld N, Nicholson E, Milner-Gulland EJ (2017). Rapid Prototyping for Decision Structuring: An Efficient Approach to Conservation Decision Analysis. Decision-Making in Conservation and Natural Resource Management.

[2] Baker CM, Campbell PT, Chades I, Dean AJ, Hester SM, Holden MH, et al. From Climate Change to Pandemics: Decision Science Can Help Scientists Have Impact. Frontiers in Ecology and Evolution. 2022;10. https://www.frontiersin.org/article/10.3389/fevo.2022.792749. Accessed 29 Mar 2022.

[3] Blomquist SM, Johnson TD, Smith DR, Call GP, Miller BN, Thurman WM (2010). Structured Decision-Making and Rapid Prototyping to Plan a Management Response to an Invasive Species. J Fish Wildl Manag..

[4] Zelner J, Eisenberg M (2022). Rapid response modeling of SARS-CoV-2 transmission. Science..

[5] National Review of Hotel Quarantine. Technical report, Australian Government Department of Health. 2020. https://www.health.gov.au/sites/default/files/documents/2020/10/national-review-of-hotel-quarantine.pdf.

[6] Hellewell J, Russell TW, The SAFER Investigators and Field Study Team, The Crick COVID-19 Consortium, CMMID COVID-19 working group, Beale R, et al. Estimating the effectiveness of routine asymptomatic PCR testing at different frequencies for the detection of SARS-CoV-2 infections. BMC Med. 2021;19(1):106. 10.1186/s12916-021-01982-x. 10.1186/s12916-021-01982-x PMC8075718 33902581

[7] Brümmer LE, Katzenschlager S, Gaeddert M, Erdmann C, Schmitz S, Bota M (2021). Accuracy of novel antigen rapid diagnostics for SARS-CoV-2: A living systematic review and meta-analysis. PLoS Med..

[8] Dinnes J, Deeks JJ, Berhane S, Taylor M, Adriano A, Davenport C, et al. Rapid, point-of-care antigen and molecular-based tests for diagnosis of SARS-CoV-2 infection. Cochrane Database Syst Rev. 2021;(3). 10.1002/14651858.CD013705.pub2. 10.1002/14651858.CD013705.pub2 PMC8078597 33760236

[9] Forde JE, Ciupe SM (2021). Quantification of the Tradeoff between Test Sensitivity and Test Frequency in a COVID-19 Epidemic-A Multi-Scale Modeling Approach. Viruses..

[10] Steyn N, Lustig A, Hendy SC, Binny RN, Plank MJ (2022). Effect of vaccination, border testing, and quarantine requirements on the risk of COVID-19 in New Zealand: A modelling study. Infect Dis Model..

[11] Grabowski F, Preibisch G, Giziński S, Kochańczyk M, Lipniacki T (2021). SARS-CoV-2 Variant of Concern 202012/01 Has about Twofold Replicative Advantage and Acquires Concerning Mutations. Viruses..

[12] Zachreson C, Shearer FM, Price DJ, Lydeamore MJ, McVernon J, McCaw J (2022). COVID-19 in low-tolerance border quarantine systems: Impact of the Delta variant of SARS-CoV-2. Sci Adv..

[13] Golding N, Shearer FM, Moss R, Dawson P, Gibbs L, Alisic E, et al. Estimating temporal variation in transmission of COVID-19 and adherence to social distancing measures in Australia. Technical report, the Peter Doherty institute for infection and immunity. 2020. https://www.doherty.edu.au/uploads/content_doc/Technical_report_15_Maypdf.pdf.

[14] Shearer FM, Moss R, McVernon J, Ross JV, McCaw JM (2020). Infectious disease pandemic planning and response: Incorporating decision analysis. PLOS Med..

[15] Duckett SJ, Sutton B (2021). On entering Australia’s third year with COVID-19. Med J Aust..

[16] Davies NG, Klepac P, Liu Y, Prem K, Jit M, Eggo RM (2020). Age-dependent effects in the transmission and control of COVID-19 epidemics. Nat Med..

[17] Wallinga J, Lipsitch M (2007). How generation intervals shape the relationship between growth rates and reproductive numbers. Proc R Soc B Biol Sci..

[18] Australian Government Department of Health and Aged Care. Public Health Laboratory Network (PHLN). 2020. https://www.health.gov.au/committees-and-groups/phln. Accessed 18 Aug 2023.

[19] Baker CM, Chades I, McVernon J, Robinson AP, Bondell H (2021). Optimal allocation of PCR tests to minimise disease transmission through contact tracing and quarantine. Epidemics..

[20] Public Health Laboratory Network, Communicable Diseases Network Australia. Joint Statement on SARS-CoV-2 Rapid Antigen Tests. Technical report, Public Health Laboratory Network. 2022.

[21] Abeysuriya RG, Sacks-Davis R, Heath K, Delport D, Russell FM, Danchin M, et al. Keeping kids in school: modelling school-based testing and quarantine strategies during the COVID-19 pandemic in Australia. Front Public Health. 2023;11. https://www.frontiersin.org/articles/10.3389/fpubh.2023.1150810. Accessed 8 Sept 2023. 10.3389/fpubh.2023.1150810 PMC10272722 37333560

[22] Hui BB, Brown D, Chisholm RH, Geard N, McVernon J, Regan DG (2021). Modelling testing and response strategies for COVID-19 outbreaks in remote Australian Aboriginal communities. BMC Infect Dis..

