# Supplementary material for: Rapid prototyping of models for COVID-19 outbreak detection in workplaces
Source: BMC Infect Dis. 2023 Oct 23;23:713. doi: 10.1186/s12879-023-08713-y (PMC10591376; doi:10.1186/s12879-023-08713-y)
Supplement: Supplementary file 1 — Additional file 1. [file 12879_2023_8713_MOESM1_ESM.pdf]

Supplementary material:
Rapid prototyping of models for COVID-19 outbreak detection in workplaces

Isobel Abell, Cameron Zachreson, Eamon Conway, Nicholas Geard,
Jodie McVernon, Thomas Waring, Christopher Baker

S1 Exponential model details

Here we provide further explanation on how the exponential model of prevalence is derived.

Let:

- P_t be the prevalence at time t. We assume this is the *number* of infectious people at time t (rather than a proportion).
- R_{eff} be the growth rate, the average number of infections arising from one infected person over their infectious period.
- g be the generation interval, the time between a person becoming infected and infecting others.
- $1/g$ be the number of "generations" in one time step. That is, we have R_{eff} infections over the generation interval, or equivalently $(R_{eff})^{1/g}$ infections per timestep.

Using these parameters, we can formulate an exponential growth model for disease prevalence:

$$P_t = P_{t-1} \times \text{growth rate in infections between time } t \text{ and } t-1$$

$$\Rightarrow P_t = P_{t-1}(R_{eff})^{1/g}$$

S1.1 Baseline parameters

In our analysis, we consider baseline parameters for the exponential model as shown in Table S1:

Parameter name	Parameter value
R_{eff}	1.1 (a conservative estimate)
Test sensitivity	85%
Test specificity	100%
Testing days per week	1 day
Generation interval	4.7 days [1]
I_0	1 person

Table S1: *Baseline exponential model parameters*

S2 Agent-Based Model details

Here we provide details on specific processes in the agent-based model. Figure S1 describes the overall structure of the ABM.

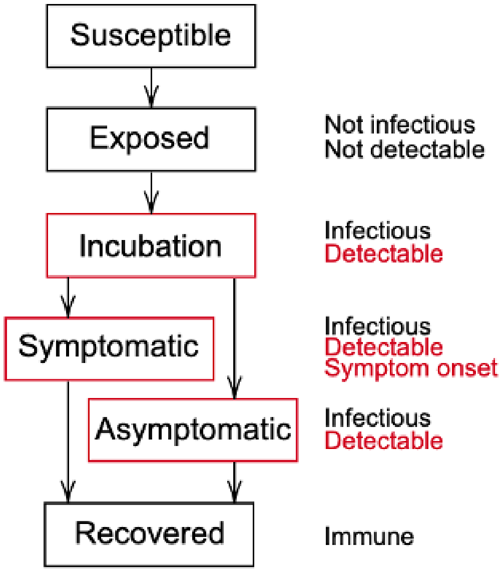

Figure S1: *Disease progression structure for the ABM*

S2.1 Probability of transmission

We consider a frequency-dependent force of infection for the agent-based model which is calculated for each person as:

$$\begin{aligned}\text{Force of infection}(t) &= \lambda(t)\\ &= \frac{\beta}{N(t)-1}P(t),\end{aligned}$$

where β is a transmission parameter calibrated for a given R_{eff}, $P(t)$ is the prevalence in the workplace on day t and $N(t)$ is the number of people in the workplace on day t. Given this force of infection, on

a given day t we assume each agent has the following probability of infection:

$$\text{Infection probability} = 1 - e^{-\lambda(t)},$$

where $\lambda(t)$ is the force of infection as given above.

S2.2 Baseline parameters

In our analysis, we consider baseline parameters for the ABM as shown in Table S2:

Parameter name	Parameter value
Duration of latent period	1 day (transmission events are unlikely to occur on the fist day after exposure [2])
Duration of incubation phase	Lognormal($\mu = 1.62, \sigma = 0.418$)
Symptomatic proportion	2/3 of infected individuals [3, 4]
Duration of symptomatic or asymptomatic phase	Uniformly distributed between 5 and 10 days [5]
Workplace size	120 people
R_{eff}	1.1
Test specificity	100%
I_0	1 person

Table S2: *Agent based model parameters*

S3 Defining testing schedules

For both the exponential and agent based models we define testing schedules by the number of days testing occurs per week. If testing occurs one day per week there are 7 possible testing schedules, two days per week there are 6 etc. until testing occurs 7 days per week, where there is only one possible testing schedule (see Table S3).

For the exponential model, as we increase the number of testing days, which schedule we choose becomes less important to the results (Figure S2). To calculate the detection probability, we take the average over all possible testing schedules.

For the agent based model, each simulation instance is assigned one of the possible testing schedules. To calculate the detection probability, we take the average over all simulation instances.

Number of testing days/week	Possible testing schedules<br>% of workforce tested<br>(Mon, Tues, Wed, Thurs, Fri, Sat, Sun)
1 day/week	(100%, 0, 0, 0, 0, 0, 0)<br>(0, 100%, 0, 0, 0, 0, 0)<br>(0, 0, 100%, 0, 0, 0, 0)<br>(0, 0, 0, 100%, 0, 0, 0)<br>(0, 0, 0, 0, 100%, 0, 0)<br>(0, 0, 0, 0, 0, 100%, 0)<br>(0, 0, 0, 0, 0, 0, 100%)
2 days/week	(100%, 100%, 0, 0, 0, 0, 0)<br>(100%, 0, 100%, 0, 0, 0, 0) . . .
⋮	⋮
7 days/week	(100%, 100%, 100%, 100%, 100%, 100%, 100%)

Table S3: *Possible testing schedules given number of testing days/week*

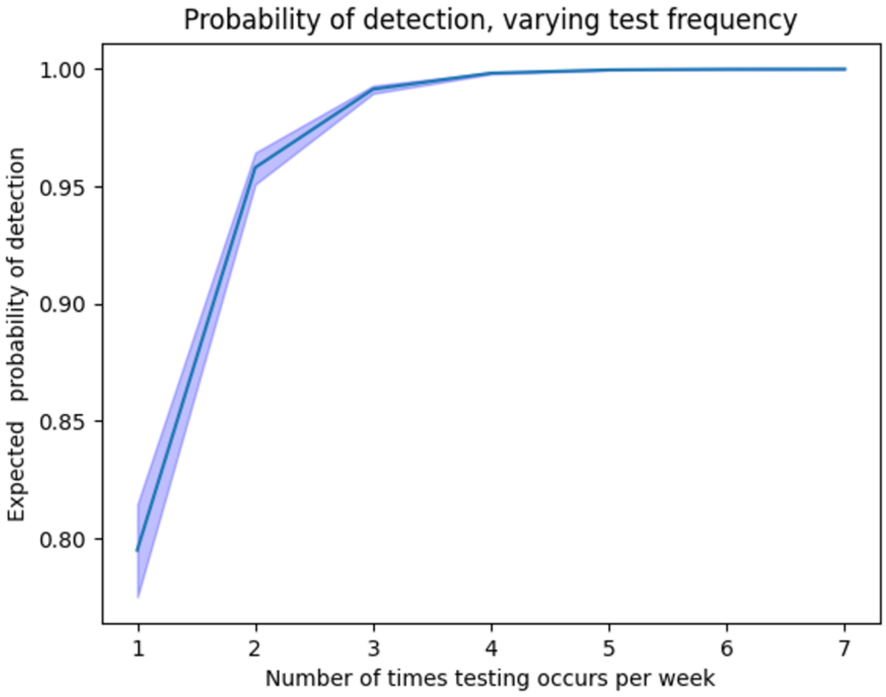

Figure S2: *Variance in testing schedules*

References

[1] Tapiwa Ganyani, Cécile Kremer, Dongxuan Chen, Andrea Torneri, Christel Faes, Jacco Wallinga, and Niel Hens. Estimating the generation interval for coronavirus disease (COVID-19) based on symptom onset data, March 2020. *Euro Surveillance: Bulletin Europeen Sur Les Maladies Transmissibles = European Communicable Disease Bulletin*, 25(17), April 2020.

[2] Luca Ferretti, Chris Wymant, Michelle Kendall, Lele Zhao, Anel Nurtay, Lucie Abeler-Dörner, Michael Parker, David Bonsall, and Christophe Fraser. Quantifying SARS-CoV-2 transmission suggests epidemic control with digital contact tracing. *Science*, 368(6491):eabb6936, May 2020. Publisher: American Association for the Advancement of Science.

[3] Cameron Zachreson, Freya M. Shearer, David J. Price, Michael J. Lydeamore, Jodie McVernon, James McCaw, and Nicholas Geard. COVID-19 in low-tolerance border quarantine systems: Impact of the Delta variant of SARS-CoV-2. *Science Advances*, 8(14):eabm3624, April 2022. Publisher: American Association for the Advancement of Science.

[4] Qiuyue Ma, Jue Liu, Qiao Liu, Liangyu Kang, Runqing Liu, Wenzhan Jing, Yu Wu, and Min Liu. Global Percentage of Asymptomatic SARS-CoV-2 Infections Among the Tested Population and Individuals With Confirmed COVID-19 Diagnosis: A Systematic Review and Meta-analysis. *JAMA Network Open*, 4(12):e2137257, December 2021.

[5] Stephen A. Lauer, Kyra H. Grantz, Qifang Bi, Forrest K. Jones, Qulu Zheng, Hannah R. Meredith, Andrew S. Azman, Nicholas G. Reich, and Justin Lessler. The Incubation Period of Coronavirus Disease 2019 (COVID-19) From Publicly Reported Confirmed Cases: Estimation and Application. *Annals of Internal Medicine*, 172(9):577–582, May 2020.
